# Eating Disorder Quality of Life Scale (EDQLS) in ethnically diverse college women: an exploratory factor analysis

**DOI:** 10.1186/s12955-018-0867-1

**Published:** 2018-03-01

**Authors:** Liya M. Akoury, Vincent Rozalski, Kimberly A. Barchard, Cortney S. Warren

**Affiliations:** University of Nevada, Las Vegas, 4505 Maryland Parkway MS 5030, Las Vegas, NV 89154-5030 USA

## Abstract

**Background:**

Extant research suggests that disordered eating is common in college women and is associated with decreased quality of life. The Eating Disorder Quality of Life Scale (EDQLS) examines impairment to disordered eating-related quality of life, but has not been validated in college women. Accordingly, the purpose of this study was to examine the reliability, validity, and factor structure of the EDQLS in a diverse sample of 971 college women.

**Method:**

Students from a large United States university completed questionnaires examining disordered eating and the EDQLS online.

**Results:**

The EDQLS demonstrated excellent internal consistency and good convergent validity with the Eating Disorder Examination Questionnaire (EDEQ). Contrary to the original 12-domain design of the EDQLS, principal component analyses suggested five factors that mapped onto the following constructs: (1) Positive Emotionality; (2) Body/Weight Dissatisfaction; (3) Disordered Eating Behaviors; (4) Negative Emotionality; and (5) Social Engagement. However, 15 of the 40 items loaded onto multiple factors.

**Conclusions:**

Total scores on the EDQLS are reliable and valid when used with diverse samples of college women, but some revisions are needed to create subscales than can justifiably be used in clinical practice.

## Background

*Disordered Eating* describes an array of symptoms characteristic of eating disorders (e.g., dietary restriction, binge eating, preoccupation with weight or shape, self-induced vomiting, excessive exercise) [1]. Disordered eating is common in women in the US [2], particularly among college women (estimates ranging 13–49%) [3], the majority of whom do not seek treatment [4].

Understanding disordered eating is particularly important for two reasons. First, disordered eating is a robust predictor of eating disorders, which have a higher mortality rate than any other mental illness [1]. Second, disordered eating is associated with a number of psychological, financial, and other costs (e.g., difficulties in social adjustment and dating) [5, 6]. Medical problems (e.g., gastrointestinal dysfunction, osteoporosis, dental problems, low fertility) are common [7] and prolonged, resulting in substantial yearly medical expenses [8] for US women.

Given these severe consequences, it is not surprising that women struggling with disordered eating experience diminished *health-related quality of life* (HRQOL), which is the impact of health problems on physical, psychological, and social domains of functioning and well-being [5]. The negative impact of eating disorders on HRQOL (i.e., *disordered eating-related quality of life*) is severe and prolonged, yet often overlooked [5, 6]. Accordingly, the inclusion of HRQOL measures in assessment and treatment of disordered eating fills an important gap in extant literature.

Reviews [6, 9] of disordered eating-specific HRQOL instruments proposed four promising candidates: (1) the Health Related Quality of Life in Eating Disorder (HeRQoLED) [10, 11]; (2) the Eating Disorder Quality of Life (EDQOL) questionnaire [12]; (3) the Quality of Life for Eating Disorders (QOL-ED) questionnaire [13]; (4) and the Eating Disorder Quality of Life Scale (EDQLS) [14]. Of these four measures, the EDQLS assesses for the broadest range of HRQOL symptoms. Specifically, the EDQLS includes items on educational/vocational functioning, future outlook, leisure activities, and individual values/beliefs. These additional domains may be developmentally salient for adolescents and emerging adults, including college women. As such, it may be a be a more robust measure for HRQOL for populations who are the highest risk for disordered eating [1]. Unfortunately, to date, the EDQLS has solely been validated in clinical samples [14, 15]. Therefore, validation of the EDQLS in non-clinical samples is a critical next step. Accordingly, the purpose of this study was to examine the psychometric properties for the EDQLS in a large, diverse sample of US college women.

## Method

### Aims

The aim of this study was to test the factor structure, reliability, and validity of the EDQLS when correlated with the Eating Disorder Examination Questionnaire [16], in a sample of ethnically diverse college women.

### Participants

Participants were 971 college women at a large university in the Southwestern US, recruited through the psychology subject pool for an online study on body image and ethnic identity [17]. Ages ranged from 18 to 66 years (*M* = 19.97; *SD* = 3.90), and body mass index (BMI) ranged from 12.93 to 51.68 kg/m^2^ (*M* = 23.43: *SD* = 4.88). Participants were 28.4% European American, 12.0% African American, 21.8% Asian American, 28.0% Latina American, 0.70% Native American, 8.4% multiracial or of a different race, 0.5% of unreported race(s).

### Measures

#### Disordered eating-related QOL

EDQLS [14] is a 40-item questionnaire designed to measure 12 domains of disordered eating-related QOL: (1) Cognitive; (2) Educational/Vocational; (3) Family and Close Relationships; (4) Relationships with Others; (5) Future Outlook; (6) Appearance; (7) Leisure; (8) Psychological; (9) Emotional; (10) Values and Beliefs; (11) Physical; (12) Eating. Items are rated on a 5-point scale, ranging from (1) Strongly Disagree to (5) Strongly Agree. Higher scores indicate higher QOL; 22 items are reverse-coded. EDQLS showed high internal consistency (Cronbach’s alpha: .96) [14]. Because we administered the EDQLS to a non-clinical sample, we replaced the words *eating disorder* with *eating habits* for the three relevant items, with the authors’ permission. In the current sample, scores ranged from 76 to 193, with a mean score of 145 (*SD* = 21.45), indicating higher QOL compared to the original clinical sample [14].

#### Disordered eating

The EDE-Q is a 31-item self-report questionnaire with four subscales: (1) Dietary Restraint (i.e., dieting, limiting or avoiding food); (2) Eating Concerns (i.e., preoccupation with food, secretiveness, and guilt about eating); (3) Shape Concerns (i.e., preoccupation with shape, body, or a flat stomach); and (4) Weight Concerns (i.e., preoccupation with weight and weight loss). Items are rated over the past 28 days on a 7-point scale (e.g., “No days” to “Every day” or “Not at all” to “Markedly”), with higher scores indicating higher levels of disordered eating symptoms [16]. The EDE-Q demonstrated excellent internal consistency in this sample (Cronbach’s alpha .94).

### Statistical analyses

We used SPSS Version 20 for all statistical analyses. Preliminary analyses of the EDQLS were as follows: (1) internal consistency, using Cronbach’s alphas; (2) corrected item-total correlation and alpha-if-item-deleted statistics to determine whether any items reduced internal consistency; (3) convergent validity with the EDE-Q, using Pearson’s correlations of each EDQLS item and total EDQLS score with the overall EDE-Q score; and (4) first principal component to determine whether all items are related to the same general construct.

To determine EDQLS factor structure, we conducted a principal components analysis with multiple factors. We used four criteria to determine the number of factors: (1) previous validation of the EDQLS [14] (suggesting up to eight factors); (2) Scree plot (suggesting five factors); (3) parallel analysis [18, 19] (suggesting five factors); and (4) the minimum average partial test [20] (suggesting four factors). Because the scree plot and parallel analysis agreed, we proceeded with a 5-factor solution. After examining several different rotations, we selected the direct oblimin rotation with delta set to − 1, because it had the smallest number of complex items, small correlations between the factors, and a high number of hyperplanar loadings. Factor loadings were considered salient if they were .30 or higher.

## Results

### Internal consistency and validity

Table 1 summarizes internal consistency and convergent validity results. Cronbach’s alpha (.93; 95% CI [.91, .93]) indicated excellent internal consistency reliability (Table 1). All items had similar values of alpha-if-item-deleted, but items 24 and 32 had poor corrected item-total correlations.

**Table 1:** Item analysis to improve internal consistency

Item	Corrected item-total correlation	Cronbach’s alpha if item deleted	Correlation with EDEQ total score	Loading on 1st principal component
1	.39	.92	−.07^*^	−.44
2	.40	.92	−.21^**^	.51
3	.26	.92	−.03	−.33
4	.27	.92	−.17^**^	.41
5	.24	.92	−.32^**^	−.45
6	.41	.92	−.29^**^	.53
7	.29	.92	−.16^**^	−.46
8	.29	.92	−.38^**^	.33
9	.32	.92	−.22^**^	−.44
10	.45	.92	−.09^**^	−.48
11	.35	.92	−.17^**^	−.47
12	.25	.92	−.20^**^	.22
13	.53	.92	−.39^**^	−.63
14	.37	.92	−.24^**^	−.52
15	.42	.92	−.51^**^	.57
16	.37	.92	−.35^**^	.53
17	.51	.92	−.39^**^	.70
18	.45	.92	−.62^**^	.53
19	.26	.92	−.24^**^	.43
20	.26	.92	−.11^**^	−.31
21	.51	.92	−.21^**^	−.56
22	.44	.92	−.21^**^	.56
23	.42	.92	−.45^**^	.57
24	.14	.92	−.01	−.21
25	.51	.92	−.65^**^	.52
26	.57	.92	−.28^**^	−.63
27	.46	.92	−.15^**^	−.45
28	.39	.92	−.18^**^	−.51
29	.50	.92	−.37^**^	.50
30	.61	.92	−.47^**^	.72
31	.56	.92	−.63^**^	.63
32	.17	.92	−.08^*^	−.27
33	.26	.92	−.36^**^	−.47
34	.43	.92	−.36^**^	.45
35	.53	.92	−.42^**^	.60
36	.36	.92	−.18^**^	−.51
37	.59	.92	−.39^**^	.71
38	.58	.92	−.68^**^	.61
39	.58	.92	−.51^**^	.61
40	.45	.92	−.24^**^	.48

Coefficient alpha for the 40-item test is .93. Coefficient alpha for the first principal component is .94. Item stems are not shown, per copyright restrictions

^*^ *p* < .05, ^**^ *p* < .01

The EDQLS had a large negative correlation with the EDEQ (*r*(969) = −.63, *p* < .001), demonstrating the convergent validity of the EDQLS: Disordered eating-related QOL is worse among those with more severe disordered eating symptoms. Items 3 and 24 had non-significant and near-zero correlations with the total score of the EDEQ, suggesting poor convergent validity for these two items.

For the first principal component, all matrix coefficients matched in valence (i.e., all positively keyed items had positive loadings; all negatively keyed items have negative loadings). Items 12, 24, and 32 had non-salient loadings (absolute values less than .30).

### Exploratory factor analysis

Principal components analysis suggested a 5-factor structure, accounting for 46.78% of EDQLS variance (25.98% by Factor 1; 8.72% by Factor 2; 4.86% by Factor 3; 3.66% by Factor 4; and 3.58% by Factor 5). When EDQLS items were grouped into five scales, based on the 5-factor structure, Cronbach’s alphas were adequate to good (.81 for Factor 1; .82 for Factor 2; .76 for Factor 3; .72 for Factor 4; and .76 for Factor 5). Tables 2 shows the rotated factor pattern matrix and our factor interpretations (items stems are not shown, per copyright restrictions).

**Table 2:** Factor Pattern Matrix Results for Rotated Factors

Item	Factor					*h* ^*2*^
	1	2	3	4	5	
26	**.70**	.20	.07	.02	.02	.62
27	**.62**	.16	**.33**	.04	.01	.52
21	**.60**	.11	.25	.19	.06	.55
13	**.60**	**.34**	.03	.01	.09	.58
36	**.58**	.05	.06	.06	.10	.44
14	**.49**	.13	.10	.16	.13	.41
11	**.46**	.08	.06	**.33**	.10	.41
28	**.39**	.06	.08	**.31**	.14	.38
32	**.30**	.02	.09	.03	.15	.15
38	.00	**.75**	.12	.15	.07	.69
25	.02	**.72**	.07	.11	.09	.58
18	.02	**.71**	.02	.07	.04	.54
31	.12	**.68**	.14	.24	.02	.62
23	.04	**.53**	.16	.09	**.36**	.51
15	.03	**.49**	**.31**	.02	.15	.47
8	.15	**.43**	**.35**	.06	.01	.36
30	.23	**.44**	.07	**.41**	.09	.61
33	.27	**.43**	.07	.14	.12	.33
5	**.33**	**.37**	.01	.03	.01	.28
29	.03	.16	**.66**	.18	.00	.58
20	**.34**	.11	**.56**	.10	.03	.42
35	.01	.29	**.58**	.08	.14	.59
39	.00	**.40**	**.54**	.09	.07	.62
34	.09	.27	**.51**	.16	.00	.45
16	.02	.20	**.39**	.27	.10	.39
24	.22	.14	**.33**	.19	.23	.25
37	.18	.24	.06	**.58**	.18	.65
4	.17	.03	.03	**.59**	.10	.39
6	.12	.13	.14	**.56**	.15	.47
12	.14	.04	**.37**	**.50**	.23	.40
17	.15	.20	.16	**.46**	.20	.54
40	.01	.05	.19	**.37**	.23	.31
1	.03	.04	.05	.06	**.76**	.58
10	.17	.04	.13	.08	**.67**	.58
3	.09	.13	.21	.13	**.57**	.40
19	.15	.16	.04	.15	**.50**	.33
22	.08	.02	.02	**.48**	**.48**	.54
7	.17	.05	.02	.08	**.46**	.33
2	.08	.00	.15	**.42**	**.43**	.48
9	**.33**	.13	.14	.02	**.34**	.34
Factor Intercorrelations						
Factor 1	1.00					
Factor 2	.14	1.00				
Factor 3	-.11	-.25	1.00			
Factor 4	.21	.27	-.12	1.00		
Factor 5	.37	.19	-.11	.23	1.00	

*Note*. *h*^*2*^ = communality. Salient factor pattern matrix coefficients are in boldface. No items were reverse-scored for this analysis. Factor 1 = Positive Emotionality; Factor 2 = Body/Weight Dissatisfaction; Factor 3 = Disordered Eating Behaviors; Factor 4 = Negative Emotionality; Factor 5 = Life Engagement

Factor 1, Positive Emotionality, assessed hopefulness about the future, self-esteem and fulfilling relationships. Factor 2, Body/Weight Dissatisfaction, assessed body image and strict dieting. Factor 3, Disordered Eating Behaviors, assessed preoccupation with food and subsequent family conflict. Factor 4, Negative Emotionality, assessed anxiety, self-doubt and other negative cognitions. Factor 5, Social Engagement, assessed positive mood and social interactions.

Interpretation of the 5-factor structure was somewhat complicated by the 15 items that cross-loaded between two factors. Interestingly, Factor 2 (Body/Weight Dissatisfaction) contained the most cross-loading items: Body image-related items (e.g., item 25) did *not* cross-load, while behavioral items (e.g., item 15) did.

## Discussion

This study examined the psychometric properties of the Eating Disorder Quality of Life Scale (EDQLS) in a large, diverse sample of college women. Results indicated excellent internal consistency and convergent validity with the EDEQ and support the use of total scores on the EDQLS in college women. Given that disordered eating is common among college women [4], evidence for the reliability and validity of the EDQLS in this population thus paves the way for additional research on HRQOL.

Our data suggested five factors: (1) Positive Emotionality; (2) Body/Weight Dissatisfaction; (3) Disordered Eating Behaviors; (4) Negative Emotionality; and (5) Social Engagement. As such, for college women, disordered eating-related QOL appears dependent on symptomatology, but also on valence of emotionality and on quality of social support. Additionally, negative emotionality likely contributes to both disordered eating-related QOL and to QOL in general, as individuals high on neuroticism are more prone to experience negative emotions and tend to exhibit more severe disordered eating symptoms [21].

However, these five factors do not match the previously proposed 12 domains [14, 15]; nor did the 12 domains combine to create five factors (i.e., items within domains loaded on different factors). Moreover, over one-third of the items loaded on multiple factors, demonstrating that even these factors are not distinct. Thus, our results do not support the use and interpretation of domain or subscale scores. However, domain or subscale scores will be essential to understanding the implications of disordered eating in clinical and community populations. We therefore urge additional research on the domains and factors of disordered eating-related quality of life, particularly through EDQLS scale revision and confirmatory factor analyses.

Per recommendations for exploratory factor analyses [22], the large sample size is a major strength, likely to produce a stable and replicable factor structure. In addition, participants were ethnically diverse college women, from one of most diverse universities in the US. As such, results may be somewhat more representative of the general US population than would be the case with traditional college samples, and may pave way for disordered eating-related QOL research in ethnically diverse populations. Accordingly, future studies should compare the EDQLS factor structure and the reliability and validity of domain scores in this and other populations. On the other hand, one limitation of our research was that we rephrased three items to include *eating habits*, rather than *eating disorder*: Participants may have reflected on their dietary preferences (e.g., veganism), rather than disordered eating symptoms. Future research should determine how the factor structure, reliability, and validity of the EDQLS are influenced by an explicit focus on disordered eating. Future research should also explore whether other personality correlates of disordered eating (e.g., perfectionism) [21] are important components of disordered eating-related QOL. Such research can inform future revisions of the EDQLS to create informative subscores to further improve this promising scale.

## Conclusion

Overall results support the use of the EDQLS total scores in ethnically diverse college women. Contrary to the original 12-domain design of the EDQLS, principal components analysis suggested that the EDQLS had five factors that mapped onto the following constructs: (1) Positive Emotionality; (2) Body/Weight Dissatisfaction; (3) Disordered Eating Behaviors; (4) Negative Emotionality; and (5) Social Engagement. We therefore recommend revision of the EDQLS so that subscale scores can be used.

## Abbreviations

BMI: Body mass index
EDE-Q: Eating Disorder Examination Questionnaire
EDQLS: Eating Disorder Quality of Life Scale
HeRQoLED: Health Related Quality of Life in Eating Disorder
HRQOL: Health-related quality of life
QOL: Quality of life
